# Safety of Vasopressor Medications through Peripheral Line in Pediatric Patients in PICU in a Resource-Limited Setting

**DOI:** 10.1155/2022/6160563

**Published:** 2022-03-31

**Authors:** Saira Abrar, Qalab Abbas, Maha Inam, Iraj Khan, Farah Khalid, Syed Raza

**Affiliations:** ^1^Department of Pediatrics, Liaquat National Hospital, Karachi, Pakistan; ^2^Department of Pediatrics and Child Health, Aga Khan University, Karachi, Pakistan; ^3^Medical College, Aga Khan University, Karachi, Pakistan

## Abstract

**Objective:**

Central venous catheter (CVC) placement in children in resource-limited settings (RLSs) can be a difficult task. Timely administration of vasopressor medications (VMs) through peripheral intravenous line (PIV) can help overcome this limitation. We aim to determine the safety of administration of vasopressor medications through PIVs in children admitted to pediatric intensive care unit (PICU) in a RLS.

**Design:**

Prospective observational study. *Setting*. An eight-bedded PICU of a tertiary care hospital. *Patients*. Children aged 1 month to 18 years admitted to the PICU. *Intervention*. None. *Measurements and Main Results*. All children (aged 1 month–18 years) who received VMs through PIV line from January 2019 to December 2019 were prospectively followed for the development of extravasation, conversion to CVC, duration of infusion, maximum dose of VMs used, maximum vasopressor inotropic score (VIS), and coadministration of vasopressor medication through PIV line. Results are presented as means with standard deviation and frequency with percentages. A total of 369 patients were included in the study, 221 (59.9%) were males, and the median age of the study population was 24 months (IQR; 6–96). Epinephrine was the most frequently used vasopressor medication (*n* = 279, 75.6%), followed by milrinone (*n* = 93, 25.2%), norepinephrine (*n* = 42, 11.4%), and dopamine (*n* = 32, 8.7%). The maximum dose of vasopressor medication was 0.25 µg/kg/min (epinephrine), 0.2 µg/kg/min (norepinephrine), 15 µg/kg/min (dopamine), and 0.8 µg/kg/min (milrinone). Extravasation was observed in 8 (2.2%) patients, while PIV line was converted to CVC in 127 (34.4%) children. Maximum dose of epinephrine, norepinephrine, VIS score, and PRISM Score was associated with conversion to CVC (*p* < 0.001), while none of them was associated with risk for extravasation.

**Conclusion:**

Vasopressor medication through PIV line is a safe option in patients admitted to the PICU.

## 1. Introduction

Vasopressor medications (VMs) are frequently used to improve the hemodynamic function in critically ill patients in emergency departments (EDs) and intensive care units (ICUs) [1] Hence, a suitable vascular access is extremely important. It has been recommended that VMs be administered via central venous catheters (CVCs) rather than peripheral intravenous lines (PIVs), except in the ED [2] The former is also commonly preferred to the latter for administering VMs. This is because of the risk of extravasation causing local tissue injury and necrosis due to the vasoconstrictive effects of vasopressor medications [1, 2].

The early initiation of vasopressor medications in shock has been associated with improved survival [3] Since CVCs require more resources and expertise, they can cause delay in initiating vasopressor medications [4, 5]. Compared to the insertion of CVCs, PIV can be easily established at any healthcare facility, which means treatment for sepsis, including the use of vasopressor drugs, can be executed without delay. This way, achieving targets of early goal-directed therapy in sepsis becomes easier, resulting in improved outcomes. Hence, initiating vasopressors via PIV can minimize the delay in treatment for shock.

In addition, the insertion of CVC is associated with various complications including mechanical and infectious [1, 2, 6] Mechanical complications may include misplacement, pneumothorax, vascular injury, and hematoma. An infectious complication is central line-associated blood stream infection (CLABSI) [7, 8].

Unfortunately, in resource-limited settings, cost and lack of adequate and skilled healthcare providers are important constraints for the routine use of CVC in pediatric intensive care units (PICUs) [6]. The number of children needing vasopressor medication infusions far exceeds the availability of CVCs. Therefore, the surviving sepsis campaign and other guidelines recommend using inotropes through PIV line as soon as possible [9] There are several factors that are necessary for the safe use of PIV access such as the requirement for frequent checks of PIV access function, prompt recognition of extravasation, and speciﬁc antidotes, e.g., phentolamine or nitroglycerin paste for extravasation, and close coordination between the healthcare providers [1, 6, 10] With more recent evidence, it can be possibly suggested that CVC might not be an immediate requirement for starting inotropic support [1, 6, 11, 12]. At present, however, there is little published data on the safety of administering vasopressor medications, especially vasopressors via PIV line in infants or children, especially from resource-limited settings [6, 13, 14]. We report the use of vasopressor medications through PIV access in the PICU in a resource-limited setting.

## 2. Materials and Methods

We conducted a prospective observational study on the use of different vasopressor medications through PIV line at the 8-bedded PICU of the Aga Khan University Hospital (AKUH) after approval from the Ethical Review Committee (2019-1310-3393). The PICU at AKUH is a multidisciplinary tertiary care PICU with around 650–750 annual admissions. The PICU is staffed with trained pediatric intensivists and fellows, as well as senior pediatric residents and critical care nurses. Only credentialed fellows and attending physicians have the privilege to place CVC. Nighttime coverage is provided by fellow and senior residents. The majority of the patients are admitted from the emergency room while patients are also admitted as spillover from cardiac ICU, operating room for postoperative care, and stepdown unit/pediatric wards.

Epinephrine is used at a concentration of 20 µg/ml, norepinephrine at 16 µg/ml, dopamine at 4 µg/ml, and milrinone at 200 µg/ml. There is generally a hesitancy in our hospital to start inotropic support through PIV line, so we made a protocol where all PIV lines are checked at least once every eight hours (or more frequently if needed) for signs of extravasation, skin and PIV integrity, and phlebitis. This is done every four hours when vasopressor medications or other high-risk medications (concentrated potassium, sodium bicarbonate, hypertonic saline, etc.) are being administered through PIV. This is documented in nursing notes and notified to the nurse team leader of the concurrent shift and physician team. If there is extravasation or phlebitis, it is reported to the hospital's adverse reporting system. For this study purpose, extravasation was classified as per Amjad et al.'s classification [15]. Extravasation is the inadvertent leakage of a vesicant solution from its intended vascular pathway (vein) into the surrounding tissue [16]. No other high-risk medications were given through the PIV line that was being used for vasopressor medication infusion. The decision to place CVC was based on the patients' condition and attending physician discretion. All children (aged 1 month to 18 years) admitted to the PICU and receiving any vasopressor medications through PIV for a minimum of four hours, from 1^st^ January 2019 to 31^st^ December 2019, were included in the study. Patients who died or were shifted from the PICU within 12 hours of the PICU stay were excluded.

The data was collected using a structured proforma, which included patient demographics, characteristics of the peripheral venous line (size, duration, and site), Pediatric Risk of Mortality III (PRISM III) score, vasopressor medication used, duration of infusion, and maximum dose administered. Medication concentration was not included as a variable since standardized concentrations for all vasopressor infusions are used in this institution. Documentation of any complications, like extravasation related to vasopressor infusion, was also collected.

VIS score was calculated using the following formula [17]:

- Inotrope score (IS) = dopamine dose (µg/kg/min) + dobutamine dose (µg/kg/min) + 100x epinephrine dose (µg/kg/min)
- Vasopressor-inotropic score (VIS) = IS + 10x milrinone dose (µg/kg/min) + 10,000x vasopressin dose (U/kg/min) + 100x norepinephrine dose (µg/kg/min)

Data on extravasation was collected from patients' charts and cross-referenced with a centralized adverse event reporting system to ensure that all complications were identified. Extravasation in PIV lines that were never used for vasopressor infusions was not included in the study. All statistical analysis was performed using the statistical software package STATA version 15.0. Qualitative variables were summarized in frequency and percentages, and quantitative variables were expressed as mean ± standard deviation (SD) or median with interquartile range (IQR) based on the normality of data. The normality of data was examined using skewness and kurtosis normality tests, and *p* value of test < 0.05 was then considered to reject the hypothesis of normal distribution. To estimate the risk ratios of conversion to CVC and extravasation for exposures including maximum doses of dopamine, epinephrine, norepinephrine, and milrinone, VIS score, and PRISM III, binomial regression was used and RRs with 95% CIs were reported. All exposure variables were used in continuous form, and there was no reference category.

## 3. Results

During the study period, a total of 810 patients were admitted and 400 children received vasopressor medication, of which 369 were included in the study. Patients were admitted to the PICU from the emergency department (*n* = 293; 79.4%), pediatric ward (*n* = 40; 10.8%), special care unit (*n* = 28; 7.6%), operating room (*n* = 7; 1.9%), and cardiac ICU (*n* = 1; 0.3%). The most common admitting diagnoses of these patients were sepsis (*n* = 107; 29.0%), respiratory illnesses (*n* = 88; 23.8%), central nervous system diseases (*n* = 60; 16.3%), hematology/oncological diseases (*n* = 44; 11.9%), cardiovascular diseases (*n* = 31; 8.4%), and others. Overall, 271 (73.4%) patients survived. The median length of hospital stay of the study population was 7 days (IQR: 4–12), and the median PRISM III score was 8 (IQR: 5–15) (Table 1).

Epinephrine was the most used vasopressor medication in our patient population, with 279 (75.6%) patients receiving epinephrine with the maximum dose of 0.25 µg/kg/min. Two or more vasopressor medications were administered simultaneously in 77 (20.9%) patients through a single PIV line. The median duration of peripheral vasopressor infusion was 24 hours (IQR: 13–48). The median VIS score was 8 (IQR: 5–10). Extravasation of the PIV access site occurred in 8 (2.2%) patients (Table 2). All of them were identified by nursing staff with prompt response using skin care and the removal of PIV line without the need for any specific treatment. The sites of PIV in these patients in whom extravasation occurred were the arm (*n* = 5) and foot (*n* = 3), respectively. The size of PIV was 20 gauge in one, 22 in four, and 24 in three patients. Two patients had grade I, four had grade II, and two had grade III extravasation. All these patients survived. There was no difference between the length of hospital stay between those who had extravasation and those who did not have extravasation (11.25 ± 9.46 and 9.14 ± 8.03 days, respectively; 95% CI 5.82–10.03, *p* = 0.55). Similarly, there was no difference in the VIS score between those who had extravasation and those who did not have extravasation (6.88 ± 2.58 and 8.76 ± 4.66, respectively; 95% CI 0.71–1.08, *p* = 0.53) (Table 3).

In 127 (34.4%) patients, PIV was converted to CVC with a median duration of 7 hours (IQR: 5–12). Reasons for conversion to CVC were persistent shock/acidosis (*n* = 32, 8.7%), requirement of persistent high inotropic support (*n* = 30, 8.1%), difficulty in maintaining PIV (*n* = 14, 3.8%), need for monitoring (*n* = 13, 3.5%), need for multiple infusions (*n* = 6, 1.6%), and physician discretion/unknown in rest of the patients (Table 2). For vasopressor medication use, an 18-gauge PIV catheter was used in 20 (5.4%), a 20-gauge catheter was used in 69 (18.7%), a 22-gauge catheter was used in 101 (27.4%), and a 24-gauge was used in 179 (48.5%) of interventions.

In univariate analysis, there were statistically significant risks of conversion from PIV to CVC in patients receiving maximum dose of epinephrine with a relative risk of 4.59 (95% CI: 3.46–6.10; *p* > 0.001) and maximum dose of norepinephrine with a relative risk of 3.04 (95% CI: 2.59–3.57; *p* < 0.001). A higher VIS score was associated with an increased risk of conversion to CVC with an RR of 1.10 (95% CI: 1.05–1.16) and a *p* value of <0.001. A similar trend was seen with the PRISM III score, which showed a statistically significant increase in the risk of conversion to CVC in patients with a higher PRISM score with a relative risk of 1.09 (95% CI: 1.05–1.13) and a *p* value of <0.001. There was no statistically significant risk of conversion to CVC with the use of maximum doses of dopamine and milrinone. The risk of extravasation was statistically insignificant in relation to the PRISM III score with an RR of 1.07 (95% CI: 0.99–1.16) (Table 3).

The RR for maximum doses of dopamine and norepinephrine is estimated as 1, which shows no association of maximum doses of dopamine and norepinephrine with extravasation. Separate models were constructed for each of the exposures (maximum dose of dopamine, epinephrine, norepinephrine, and milrinone, VIS score, and PRISM III) with each outcome including conversion to CVC and extravasation.

## 4. Discussion

This study demonstrated that administration of vasopressor medication via PIV access in critically ill children admitted to the PICU is associated with a low incidence rate of extravasation injury and is, therefore, a safe alternative route in resource-limited settings or before CVC access can be safely obtained. The majority of our study population had sepsis with or without septic shock or respiratory illnesses as admitting diagnosis and were admitted from ED. The risk of conversion to CVC was highest with maximum doses of epinephrine and norepinephrine. High VIS and PRISM scores were also factors associated with increased conversion of PIV to CVC. The most used vasopressor agent in our study was epinephrine, although most adult and pediatric studies indicated norepinephrine and dopamine, respectively, as the most used agent [2, 13, 23]. The median time to CVC conversion in our study was 7 hours compared to 3 hours in a study conducted in the US by Patregnani et al. Extravasation had significant association with the PRISM III score. The PRISM III score association with extravasation risk revealed a significant *p* value (*p* = 0.005) with 95% CI crossing 1 (0.99–1.16). The RR for this is 1.07, so it can be said that this has limited clinical applicability based on our data. This is also based on univariate analysis, so it is potentially confounded by many other factors. While it did not have any statistically significant correlation to any of the other factors, e.g., maximum doses of vasopressor medication or VIS scores, the incidence rate of extravasation injury in this study cohort is 2.2%, which is similar to a previously reported pediatric study by Kumar et al. where they studied short-term use of PIV in their PICU which showed a 1.5% rate of extravasation [6, 13]. The median duration of vasopressor medication use through PIV was 24 hours, which is higher than the median duration seen in a previous similar study by Patregnani et al. (9 hours) [13]. Since the duration of vasopressor medication use in our study is longer and is not associated with significant complications, it can be extrapolated that vasopressor medication infusions through PIV access can be safely given for longer durations. Future studies that specifically test/observe the association of duration of vasopressor medication use via PIV access and complications resulting from it would substantiate this finding even further.

Our study echoes the conclusion of a similar study conducted in the adult population, which showed that the risk of developing complications with vasopressor medication infusions through PIV line was negligible and not clinically significant [25].

Vasopressor medication support is an important part of the initial resuscitation of septic and other shocks as well as any critical illness. The safety associated with the use of peripheral access, as opposed to central access, to administer vasopressor medications has become a necessary topic to investigate, especially in a resource-limited setting. Inotropic support is an important component of early goal-directed therapy (EGDT) in septic shock, and EGDT has been shown to be shown to be associated with improved outcomes in these patients [9, 18, 19]. One study in adult septic shock has shown that for every 1-hour delay in norepinephrine infusion for septic shock, mortality increased by 5.3% [20]. One important factor that may contribute to a delay in the administration of vasopressor medication is the reluctance of healthcare workers to give these medications via PIV access due to the association of many complications, e.g., skin necrosis and limb ischemia; another factor is the lack of expertise and resources available for establishing CVC access in resource-limited settings [21, 22].

Before the introduction of the VMs through the PIV, a common indication for CVC insertion in our PICU was the perception that VMs could only be administered through CVC, to avoid local tissue injury because of extravasation of VMs through the PIV. Our results indicate that vasopressor medication use is not an automatic indication for CVC insertion. In a resource-limited setting with a paucity of highly trained healthcare workers, this study shows a safe alternative to vasopressor medication infusions via CVC.

### 4.1. Strengths and Limitations

Our study describes a comprehensive report on the use of vasopressor medications through PIV access in severely ill/injured children admitted to the PICU. There are several limitations to our study. Our study has been conducted in a single institute in a heterogenous study population admitted to the PICU, so generalizability may be limited. We did not include any details on how extravasation injury may have affected healthcare resource utilization. Hence, the study's applicability is limited.

Our data analysis included only a univariate analysis in terms of studying the relative risk of sustaining extravasation injury and conversion to CVC. Another limitation is that this study did not have a comparison group. There are several elements that may have allowed the safe use of vasopressor medications via PIV access, which are repeated examination of access by staff and quick identification of and measures to counter extravasation [1].

## 5. Conclusion

Administration of vasopressor medication via PIV access is shown to be safe with good adherence to safety protocols applied by the patient's bedside. Clinicians should not regard the use of vasopressor medication as an automatic indication for central venous access.

## Figures and Tables

**Table 1:** Clinical characteristics of study population (*n* = 369).

Characteristics		N (%)
Gender	Male	221 (59.9%)
	Female	148 (40.1%)
Median age (months) (interquartile range (IQR))		24 (6–96)
Median weight (kg) (IQR)		10 [6–24]
WAZ (weight for age Z-scores)	Underweight	100 (36.4%)
	Normal	175 (63.6%)
Admission source	Emergency room	293 (79.4%)
	Ward	40 (10.8%)
	SCU	28 (7.6%)
	CICU	1 (0.3%)
	Operating room	7 (1.9%)
Admission diagnosis	Sepsis	107 (29%)
	Respiratory disease	88 (23.8%)
	CNS	60 (16.3%)
	Hematology/oncology	44 (11.9)
	CVS	31 (8.4%)
	Renal	10 (2.7%)
	GI	8 (2.2%)
	Miscellaneous	21 (4.3%)
PRISM III (median-IQR)		8 [5–15]
Outcome (survival)		271 (73.4%)
Median length of hospital stay (days) (IQR)		7 (4–12)

IQR = interquartile range, SCU= special care unit (high-dependency unit), CICU = cardiac intensive care unit, CNS = central nervous system, and GI = gastrointestinal system.

**Table 2:** Vasopressor use and peripheral catheter data (*n* = 369).

Variables		
Vasopressor medication used	Dopamine	32 (8.7%)
	Epinephrine	279 (75.6%)
	Norepinephrine	42 (11.4%)
	Milrinone	93 (25.2%)
Maximum dose of vasopressor medication used (µg/kg/min)	Dopamine	15
	Epinephrine	0.25
	Norepinephrine	0.2
	Milrinone	0.8
Median duration of vasopressor medication used (hours) (IQR)		24 (13–48)
Vasopressor inotropic score	<5	147 (39.8%)
	5–10	167 (45.3%)
	11–15	29 (7.9%)
	16–20	20 (5.4%)
	>20	6 (1.6%)
Vasopressor inotropic score (median; IQR)		8 [5–10]
Coadministration of vasopressor medication		77 (20.9%)
Size of peripheral IV catheter	18	20 (5.4%)
	20	69 (18.7%)
	22	101 (27.4%)
	24	179 (48.5%)
Conversion to central venous catheter (CVC)		127 (34.4%)
Reason for CVC conversion	Persistent high inotropic support	30 (8.1%)
	Difficulty to maintain PIV	14 (3.8%)
	Persistent acidosis	32 (8.7%)
	Need for multiple infusions	6 (1.6%)
	Monitoring	13 (3.5%)
	Unknown	30 (8.1%)
Time to CVC; hours (median; IQR)		7 [5–12]
Extravasation		8 (2.2%)

IQR=interquartile range.

**Table 3:** Univariate analysis.

Factors	Outcome			
	Conversion to CVC		Extravasation	
	RR (95% CI)	*P* value	RR (95% CI)	*P* value
Maximum dose of dopamine	0.98 (0.93, 1.03)	0.503	1	
Maximum dose of epinephrine	4.59 (3.46, 6.10)	0.0001	1.21 (0.49, 2.95)	0.310
Maximum dose of norepinephrine	3.04 (2.59, 3.57)	0.0001	1	
Maximum dose of milrinone	1.34 (0.74, 2.42)	0.380	0.22 (0.00, 18.53)	0.247
VIS score	1.10 (1.05, 1.16)	0.0001	0.87 (0.71, 1.08)	0.531
PRISM III	1.09 (1.05, 1.13)	0.0001	1.07 (0.99, 1.16)	0.005

## Data Availability

Data can be shared upon reasonable request to the corresponding author.
